# Experimental Demonstration of Supervised Learning in Spiking Neural Networks with Phase-Change Memory Synapses

**DOI:** 10.1038/s41598-020-64878-5

**Published:** 2020-05-15

**Authors:** S. R. Nandakumar, Irem Boybat, Manuel Le Gallo, Evangelos Eleftheriou, Abu Sebastian, Bipin Rajendran

**Affiliations:** 1 IBM Research – Zurich, 8803 Rüschlikon, Switzerland; 2 New Jersey Institute of Technology, Newark, NJ 07102 USA; 3 Ecole Polytechnique Federale de Lausanne (EPFL), 1015 Lausanne, Switzerland; 4 King’s College London, Strand, London WC2R 2LS United Kingdom

**Keywords:** Electrical and electronic engineering, Nanoscale devices

## Abstract

Spiking neural networks (SNN) are computational models inspired by the brain’s ability to naturally encode and process information in the time domain. The added temporal dimension is believed to render them more computationally efficient than the conventional artificial neural networks, though their full computational capabilities are yet to be explored. Recently, in-memory computing architectures based on non-volatile memory crossbar arrays have shown great promise to implement parallel computations in artificial and spiking neural networks. In this work, we evaluate the feasibility to realize high-performance event-driven *in-situ* supervised learning systems using nanoscale and stochastic analog memory synapses. For the first time, the potential of analog memory synapses to generate precisely timed spikes in SNNs is experimentally demonstrated. The experiment targets applications which directly integrates spike encoded signals generated from bio-mimetic sensors with in-memory computing based learning systems to generate precisely timed control signal spikes for neuromorphic actuators. More than 170,000 phase-change memory (PCM) based synapses from our prototype chip were trained based on an event-driven learning rule, to generate spike patterns with more than 85% of the spikes within a 25 ms tolerance interval in a 1250 ms long spike pattern. We observe that the accuracy is mainly limited by the imprecision related to device programming and temporal drift of conductance values. We show that an array level scaling scheme can significantly improve the retention of the trained SNN states in the presence of conductance drift in the PCM. Combining the computational potential of supervised SNNs with the parallel compute power of in-memory computing, this work paves the way for next-generation of efficient brain-inspired systems.

## Introduction

In recent years, deep learning algorithms have become successful in solving complex cognitive tasks surpassing the performance achievable by traditional algorithmic approaches, and in some cases, even expert humans. However, conventional computing architectures are confronted with several challenges while implementing the multi-layered artificial neural networks (ANNs) used in these algorithms, especially when compared against the approximately 20 W power budget of the human brain. The inefficiencies in the von Neumann architecture for neural network implementation arise from the high-precision digital representation of the network parameters, constant shuttling of large amounts of data between processor and memory, and the ensuing limited computational parallelism and scalability. In contrast, the human brain employs billions of neurons that communicate with each other in a parallel fashion, through dedicated, analog, and low-precision synaptic connections. The spike-based data encoding schemes used in these biological networks render the computation and communication asynchronous, event-driven, and sparse, contributing to the high computational efficiency of the brain.

The size and complexity of artificial neural networks are expected to grow in the future and has motivated the search for efficient and scalable hardware implementation schemes for learning systems^1^. Spiking neural networks (SNNs) are being explored for efficient learning systems^2^, especially for energy and memory-constrained embedded applications, as they mimic computational principles of the brain such as spike encoding of data, event driven computation, and temporal integration. Application specific integrated circuit (ASIC) designs such as TrueNorth from IBM^3^ and Loihi from Intel^4^ that implement SNN dynamics have been successful in demonstrating two to three orders of magnitude energy efficiency gain by using such sparse, asynchronous, and event-driven computations. However, these fully digital implementations use area expensive static random access memory (SRAM) circuits for synaptic weight storage which could potentially limit the amount of memory that can be integrated on-chip and the scalability of these architectures.

**Figure 1:**
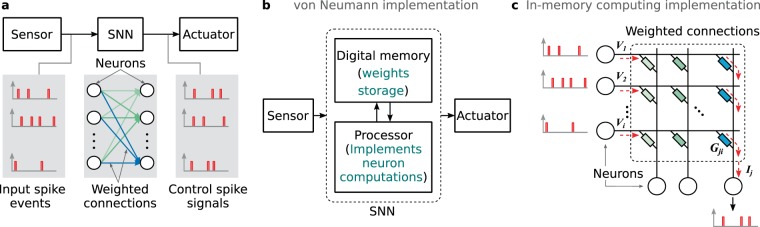
Spiking neural network in different architectures. (**a**) A single layer SNN that interface with a sensor to translate an input set of spike trains to an output set of spike trains which act as control signals for an actuator. The SNN consists of neurons with weighted connections. (**b**) A von-Neumann architecture implements the neuron dynamics in a processor and the connection weights are stored in a physically separate digital memory. Here, processing the asynchronous spike streams in the SNN requires frequent access to the weight memory. (**c)** An in-memory computing architecture uses a crossbar array with analog memory devices at its junctions storing the connection weights in its conductance. Dedicated neuron circuits are implemented at the crossbar periphery. The input spike streams can be directly applied as voltage pulses to the crossbar word lines and the weighted summation is obtained as currents along the bit lines. These currents are processed by the neuron circuits to generate output spikes.

Consider an application where an SNN is tasked with learning to generate a certain set of control signal streams encoded using precisely timed spikes based on spike inputs from a neuromorphic sensor (Fig. 1a). Artificial retina^5^ and silicon cochlea^6^ which employ spike encoding schemes for visual and auditory signals are interesting examples for such neuromorphic sensors. A von Neumann implementation of the SNN will have to access the weights from a digital memory for each input spike event and transfer them to a physically separate processor to implement the neuron dynamics (Fig. 1b). This constant shuttling of data and the resulting memory bottleneck has made hardware implementations of neural networks energy expensive^7^. In-memory computing is a promising alternative which co-locates processing and storage. For example, crossbar arrays of analog non-volatile memory devices can perform weighted summation of its word line voltages in parallel using the device conductances and the results are available as currents at its bit lines^8–11^ (Fig. 1c). This memory architecture performs computations using a combination of Ohm’s law and Kirchhoff’s law to reduce matrix-vector multiplications to *O*(1) complex operations. SNNs processing asynchronous events in time can significantly benefit from such an on-chip computational memory that could store the synaptic weights in the device conductance values and provide dedicated connectivity patterns to process parallel events in real-time.

The task of generating precisely timed spikes requires a supervised training algorithm. The discontinuity in the neuron responses during spike events in SNNs prevent the direct application of traditional gradient descent based backpropagation algorithms to train SNNs^12^. Alternatively, training can be performed in an equivalent ANN which is then converted to an SNN^13–15^ or pseudo derivatives can be used in place of non-differentiable spike responses to perform gradient descent training in SNNs^16–18^ However, most of these approaches are designed to generate desired spike rates rather than spike times at the output. Recently, several approximate spike time based supervised training algorithms have been proposed of varying computational complexity that has demonstrated various degrees of success in benchmark problems in machine learning^19^. Among these, SpikeProp^20^ is designed to generate single spikes and Tempotron^21^ uses a non-event driven error computation. SLAYER accumulates error gradients for every time step of SNN simulation during training and is hence computationally expensive^22^. Among the event driven update rules ReSuMe^23^ and NormAD^24^, the latter show higher convergence and achieved high classification accuracy for the MNIST hand-written digit recognition benchmark^25^.

Our work experimentally evaluates the task of supervised training to generate precisely timed spikes when the SNN synapses are implemented using phase-change memory (PCM) devices. We use a single layer SNN whose synapses are realized using 177,000 PCM devices from a prototype chip. We exploit the gradual and cumulative conductance modulation of PCM devices and the NormAD supervised training algorithm to achieve the spike generation task. PCM is a mature non-volatile memory technology that has demonstrated potential for both SNN^26–32^ and ANN^30,33–35^ implementations. However, the device exhibits certain non-ideal characteristics that are typical to most nanoscale memories, such as limited precision, stochasticity, non-linearity, as well as drift of the programmed conductance states with time. The impacts of the PCM non-idealities have been evaluated before in standard classification tasks using both ANNs^36–39^ and SNNs^40–43^. The previous studies on SNNs investigate classification tasks based on the neuron spiking the most or first-to-spike neuron. Our work differs from the previous investigations as we evaluate the effects of PCM non-idealities in training an SNN to generate spikes at precise times. This problem is more sensitive to device non-idealities because it involves the more challenging task of training each output neuron to spike at precise times, rather than just promoting single output neuron to spike while suppressing spikes from others as in the case of a classification task. Finally, we experimentally demonstrate that the SNN response can be maintained over a period of 4 days on PCM hardware in the presence of conductance drift using a global scaling technique without significant loss in spike time accuracy.

## Results

### Phase-change memory device

**Figure 2:**
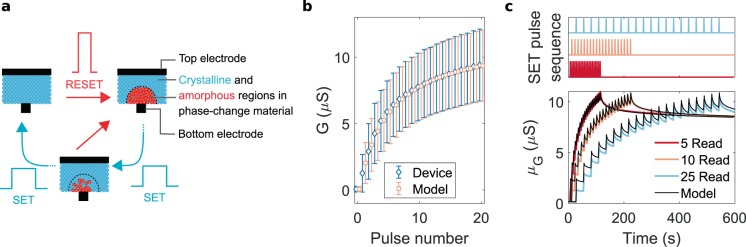
Phase-change memory characteristics. **(a)** Phase-change memory devices have a phase change material sandwiched between a top electrode and a narrow bottom electrode. In a mushroom like device structure as in the figure, application of a RESET pulse creates an amorphous dome around the narrow bottom electrode. The crystalline state of the material can be reversed by the application of SET pulses which induce crystal growth and gradually increases the conductance of the device. (**b)** Non-linear conductance evolution characteristics of PCM in response to a sequence of SET pulses with 90 *μ*A amplitude and 50 ns width. The programming statistics are obtained from 10,000 devices integrated in a 90 nm prototype chip. Error bars show one standard deviation. Corresponding model response is also shown. (**c)** Conductance evolution of PCM when programmed with 20 SET pulses of different delays. SET programming pulses are delayed by inserting different number of read pulses in between.

For our on-chip training experiment, we used a prototype chip containing more than one million doped-Ge_2_Sb_2_Te_5_ (GST) based PCM devices fabricated in 90 nm CMOS technology node^44^. In this section, we present the conductance evolution behavior of the devices in response to programming, and a statistical model developed to capture it. The PCM device dielectric, GST, has low resistivity in its poly-crystalline state and a high resistivity in its amorphous phase. We use the ability to reversibly transition between these two phases to update the conductance of PCM based synapses. The device structure has the dielectric sandwiched between a top electrode and a narrow bottom electrode (Fig. 2a). The as-fabricated dielectric is in the crystalline phase. An amorphous region can be created around the narrow bottom electrode via heat-induced melting and subsequent quenching using a short current pulse, resulting in a low conductance device state. The conductance can be gradually increased by a sequence of SET pulses, which increases the device temperature to its crystallization regime and reduce the amorphous volume via crystal growth. This conductance increment process, called SET, can be gradual and accumulative using suitable current amplitudes. However, the melt-quench process called RESET which creates the amorphous region is observed to be non-accumulative and abrupt.

Figure 2b shows the conductance evolution statistics from 10,000 devices initialized to around 0.1 *μ*S and subjected to 20 SET pulses of 90 *μ*A amplitude and 50 ns width. The mean of the conductance change per subsequent programming pulse decreases and its standard deviation increases causing a non-linear update behavior. We analyzed the state-dependent nature of the conductance change in the devices and developed a statistical model capturing the stochastic conductance evolution behavior^45^. The model response is also shown in Fig. 2b. In addition, PCM exhibits conductance drift over time which is attributed to the structural relaxation of the programmed state^46–48^. Our PCM model incorporates this conductance drift and read noise (Supplementary Note 1). The drift in PCM is observed to re-initiate after each programming event^39,45^. To ensure that the model captures the drift re-initialization in PCM properly, we performed an experiment where the devices are applied with SET pulse sequences with different time delays. This emulates the situation in neural network training, where a different set of synaptic memory devices will be programmed at different time instances and may drift by different rates. Figure 2c shows that the model captures the conductance drift behavior in PCM reliably.

### SNN learning experiment

**Figure 3:**
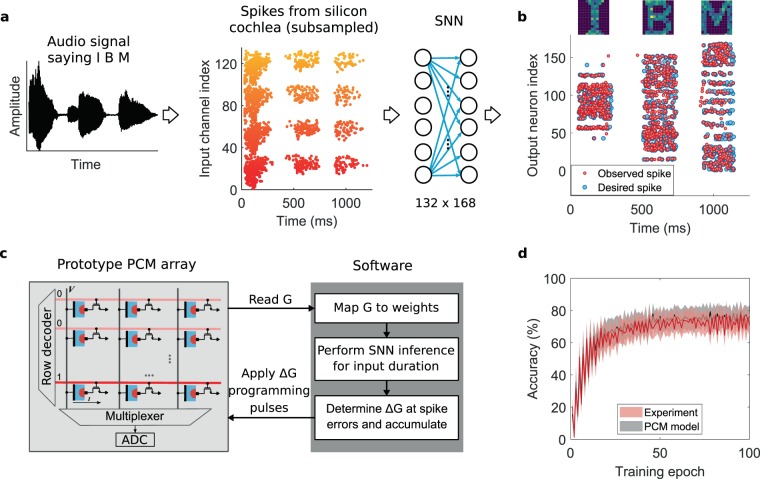
SNN training experiment using PCM devices. (**a**) The audio signal is passed through a silicon cochlea chip to generate spike streams. These spike streams are sub-sampled to generate the input training patterns for the SNN. The single-layer SNN has 132 inputs fully connected to 168 output LIF neurons. (**b**) The desired output spike streams are generated by a Poisson random process whose inter-arrival rate is made proportional to the intensities of 14 × 12 pixel images of characters I, B, and M. A raster plot of the desired spike trains along with the character images used to generate them are shown. The observed spike trains at the end of 100 epochs of training experiment using the PCM devices are also shown. (**c**) An illustration of our hardware/software training simulation setup. All the weight updates are applied directly to the weights stored in the prototype PCM array. The programmed values are read and used to implement SNN dynamics and compute weight updates in software. (**d**) Accuracy as a function of training epochs from the experiment using on-chip PCM devices. Each synapse was realized using 8 PCM devices in a differential configuration. Accuracy is defined as the fraction of the spike events in the desired pattern corresponding to which a spike was generated from respective output neurons within a certain time interval. The lower bound of the shaded lines correspond to 5 ms interval, the middle line to 10 ms and the upper bound to 25 ms. The corresponding training simulation using the PCM model shows excellent agreement with the experimental result.

This section presents the experiment where PCM device based synapses are trained to generate streams of spikes at desired time instances. We use a single layer SNN with leaky-integrate-and-fire (LIF) output neurons. For illustrative purpose, a Silicon cochlea chip^6^ is used to generate the input to the network which translates a spoken audio signal ‘IBM’ (Eye..Bee..Em) into 132 spike streams (see Methods). The desired SNN output spikes are created by tuning the spike arrival rates of the 168 output neurons to be proportional to the pixel intensities of 14 × 12 pixel images of the characters ‘I’, ‘B’, and ‘M’ using a Poisson random process. The audio signal, raster plot of the input spike streams, and the SNN network are illustrated in Fig. 3a. The corresponding desired output spike streams are shown in Fig. 3b.

The 22,176 synapses of the single-layer SNN are realized using 177,408 PCM devices, whereby 8 PCMs represent one synaptic weight. Multiple PCMs were used per synapse to increase the conductance range available to a synapse for a given conductance update size. Due to the gradual SET and abrupt RESET transition characteristics of the device, each synapse is implemented using a differential configuration^40^, i.e., for each network weight between an input neuron *i* and an output neuron *j*, ${W}_{ji}=\beta ({G}_{pji}-{G}_{nji})$, where *G*_*pji*_ and *G*_*nji*_ are realized as the total conductance of half the PCM devices allotted per synapse. *β* is a scaling factor. This allows both the potentiation and depression of the synaptic conductance via SET pulses applied respectively to one of the *G*_*pji*_ and *G*_*nji*_ devices chosen cyclically^30^. An eventual hardware implementation of the computational memory can be designed to perform the weighted summation of the input spikes using a crossbar array with the PCM devices with access transistors at the junctions as shown in Fig. 1c. Due to the serial interface to the prototype chip hosting the PCM array used for this experiment, all the device conductance values were read using constant voltage pulses for each training epoch, mapped to corresponding weight values in software and were used to determine the SNN response for the input pattern in the experiment (Fig. 3c). In an eventual hardware implementation of the SNNs, where the spike information from one neuron layer to the other is transferred using fixed amplitude rectangular pulses, the fixed voltage read pulses in our current implementation already captures PCM non-linear conductance behavior. However, we neglect the effect of the ohmic drop in the crossbar lines in our calculations.

Let ${S}_{i,in}(t)={\sum }_{{t}_{i}}\delta (t-{t}_{i})$ represent the spike stream from input neuron *i*. The weight matrix, mapped using the PCM conductances, perform the weighted summation of input spike streams $({\sum }_{i}{W}_{ji}{S}_{i}(t))$. This net spike input to an output neuron is convolved with a synaptic current kernel mimicking the response of a biological synapse, ${I}_{ker}(t)=({e}^{-t/{\tau }_{1}}-{e}^{-t/{\tau }_{2}})u(t)$, and the resulting current is integrated by the LIF neuron. Here $u(t)$ is the Heaviside step function, ${\tau }_{1}=5$ ms, and ${\tau }_{2}=1.25$ ms. Integration of the total input current increases the membrane potential of the LIF neuron model and when this voltage exceeds a threshold, an output spike is issued. The membrane potential exceeding the threshold is reset to resting potential and is prevented from further input integration for 2 ms, typically known as the refractory period (see Methods). We gradually adjust the PCM conductance values to match the observed output spike trains to the desired ones using a supervised training algorithm called normalized approximate descent (NormAD)^24^. In NormAD, weight updates are computed at events when there is an observed or desired spike at any of the output neurons and they do not coincide with each other. Therefore, the error at an output neuron *j* is defined as ${e}_{j}(t)={S}_{j,des}(t)-{S}_{j,obs}(t)$ and updates to the afferent weights are computed whenever ${e}_{j}(t)\ne 0$ as,

1 $$\varDelta {W}_{ji}(t)=\eta \times sgn({e}_{j}(t))\times \frac{{\hat{d}}_{i}(t)}{\Vert \hat{d}(t)\Vert }dt$$

where $\eta$ is the learning rate. ${\hat{d}}_{i}(t)$ is obtained by convolving the input spike stream ${S}_{i,in}(t)$ with ${I}_{ker}(t)$ and an approximate impulse response of the LIF neuron (see Methods). ${\hat{d}}_{i}(t)$ is normalized using the 2-norm of ${\hat{d}}_{i}(t)$ from all the input neurons so that the magnitude of the total weight update at any spike error is fully determined by the learning rate. Equation (1) suggests a potentiation at the desired spike and depression at an observed spike unless they coincide. The weight updates could be directly applied to the device at each time instance of the error. However, to reduce the total number of programming events to the devices and to avoid a faster conductance saturation from the repeated SET programming to the PCM devices, the weight updates at spike events were accumulated over the input pattern duration and were applied to the devices at the end of each presentation of the entire training pattern (a training epoch). The Δ*W*_*ji*_s $({\sum }_{{e}_{j}(t)\ne 0}\varDelta {W}_{ji}(t))$ were converted to desired conductance changes using the scaling factor $\beta$ and were mapped to programming pulses of suitable amplitude (see Methods). At the end of each training epoch, these programming pulses were applied to corresponding PCM devices blindly without verifying if the observed conductance change matches the desired update. This blind programming scheme (without expensive read-verify) is expected to be the norm of computational memory-based learning systems in the future. In this study, we experimentally evaluate the potential of analog PCM conductance to accurately generate spikes at the desired times in the presence of such noisy updates.

The training sequence was repeated for 100 epochs. The training set consists of 132 input spike trains lasting 1250 ms and the corresponding 168 desired output spike trains. The entire data set was applied to the network for each training epoch and the weight updates were computed at spike errors at the output neurons. The total weight updates accumulated over the training pattern were applied to the PCM at the end of training using blind programming pulses. All the PCM conductance values were read from the array, supplied to the SNN software simulator, and the training epoch was repeated. The conductance values read include the effects of stochastic programming, read noise, and conductance drift between the training epochs. We also implemented an early stop to the neurons that generated the desired spike streams within 0.5 ms, by which we excluded the synapses connected to those neurons from further conductance updates.

### Training performance

In our illustrative example, the SNN is tasked with generating 987 spikes at the output layer when it is excited by the input spikes from the audio input. To evaluate the performance, we define spike generation accuracy as the fraction of the desired output spikes which have an observed spike within a certain time interval. At the end of each training epoch, for each desired spike, the corresponding observed spike is determined using a nearest neighbor search and the resulting percentage accuracies determined for three different tolerance time intervals are shown in Fig. 3d. In the line plot of accuracies with shaded bounds, the lower bound, middle line, and the upper bound correspond to spike time tolerance intervals of 5 ms, 10 ms and 25 ms, respectively. Note that the average output spike rate for each of the character duration was less than 20 Hz corresponding to an inter-arrival time of 50 ms, and the task of the network is to create spikes each one of which can be unambiguously associated with one of the target spikes. We observe that the trained SNN generated 85.7% of the spikes within the 25 ms error tolerance. A raster plot of the spikes observed from the SNN trained in the experiment is shown in Fig. 3b as a function of time along with the desired spikes.

We also used the PCM conductance update model presented in Section 2.1 to simulate the training experiment fully in software. The model-based training simulation following the same weight update scheme captures experimental observations reliably (Fig. 3d) (see also Supplementary Fig. 1). We use this model to study the effect of conductance drift and stochasticity of PCM in the training performance.

### Effects of non-ideal synapse

**Figure 4:**
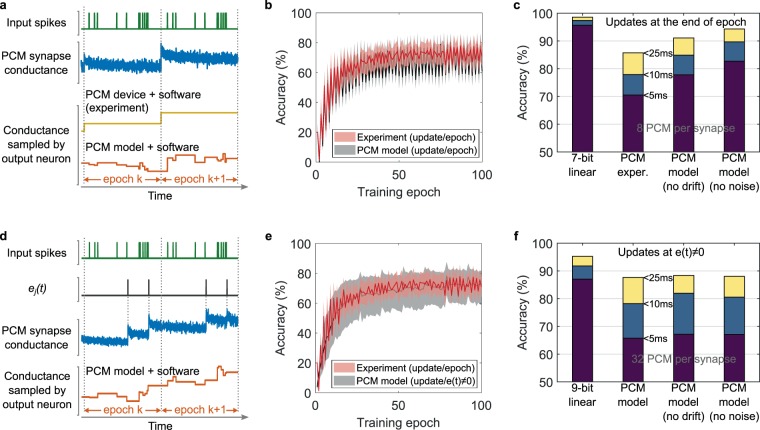
Effects of non-ideal synaptic device behavior. **(a**) For an exemplary input spike pattern (green), PCM synaptic conductance evolution (blue) over time obtained from the model, over two consecutive training epochs, *k* and *k* + 1. At the beginning of each epoch, the synaptic conductance values are updated based on the learning rule. While we read the conductance values only once at the beginning of each epoch in the experiment (yellow), we used the model to study the effect where the instantaneous value of the conductance is sampled by the output neuron at every input spike (as in the example curve in orange). (**b)** The accuracy from the model-based training is compared with those from the experiment. The lower bound of the shaded lines correspond to 5 ms tolerance interval, the middle line to 10 ms and the upper bound to 25 ms. The model uses the instantaneous synapse conductance at the times of input spikes for LIF neuron integration compared to the experiment which uses the value read from the hardware at the beginning of each epoch. (**c**) The maximum observed accuracy for different synapse models for the SNN. The top of violet, blue, and yellow bars indicate the percentage of output spikes within 5 ms, 10 ms, and 25 ms tolerance intervals, respectively. The x-axis label of each bar represents the SNN synapse model/precision used during training. (**d**) Illustration of a model-based training which implements spike-triggered synapse conductance update. The PCM synapse conductance (blue) is updated at times ${e}_{j}(t)\ne 0$ (black). The updated conductance values are sampled by the output neuron at the times of input spikes. (**e)** The accuracy from model-based training with the spike-triggered conductance update is compared with the experiment. Model-based training uses 32 PCM per synapse. (**f**) The maximum observed accuracy for different synapse models for the SNN with spike-triggered conductance updates. The PCM model-based training uses 32 PCM per synapse.

Figure 4a shows at the top, one of the input spike streams for two consecutive training epochs. An illustration of conductance evolution of a PCM based synapse, connecting the input spikes to an output neuron, based on our PCM model is also shown (blue line). The conductance evolution shows the drift, programming noise, and read noise. Note that the conductance is updated only at the end of each epoch. The hardware experiment reads the device conductance values once after each update, and this value is used by the neuron for its computations during the epoch. To study the effects of conductance drift and read noise, a PCM model-based training simulation was performed where the conductance values of the PCM devices were sampled at every input spike as shown by the orange curve in Fig. 4a. Though incorporating the PCM temporal evolution within the simulation of each training epoch caused slightly larger accuracy fluctuations between subsequent epochs, it maintained experiment comparable accuracy (Fig. 4b).

The SNN when trained using double-precision floating-point (FP64) synapses achieved above 99% accuracy for the spike time tolerance of 25 ms. In comparison, the PCM device based experiment achieves 85.7% for the same tolerance interval. The accuracy drop is mostly attributed to the device non-idealities such as nonlinear and stochastic conductance response as well as temporal evolution of device conductance. To understand the impact of individual device non-idealities, we conducted simulations with various device models. First, we simulated training using a linear 7-bit device, whose number of weight levels are approximately comparable to our 8 PCM-based synapse. This is shown to perform the same task with 98.5% accuracy (see Fig. 4c), indicating that the aforementioned factors are indeed limiting the training performance. Next, we used the non-linear PCM model with conductance drift turned off. Here, the device stochastically programmed to a state remains in that state, except for read noise induced jitter. In the absence of drift, the accuracy increased to approximately 91%. Further, the non-linear PCM model was modified to have zero programming noise and read noise, while incorporating conductance drift. In this case, the synapses which successfully generated the desired spikes will undergo further conductance drift and hence will reduce the convergence rate. Hence it was necessary to continue the training for all the neurons without an early stop to maintain/improve the training accuracy. We achieved 92.4% accuracy in this scenario (final bar in Fig. 4c). Conductance saturation from the repeated SET programming in the differential synapse is another potential factor that could limit the training accuracy^40^. However, in our 100 epochs of training, the average number of programming events per PCM in the experiment was less than 5 and hence the effect of saturation can be neglected. In short, the programming noise seems to be the dominant factor limiting the training accuracy. Also, we observe that continuing the training for the converged neurons can recover some of the accuracy loss due to the temporal drift behavior.

In the experiments and simulations so far, a mixed-precision training approach was used whereby the desired weight changes are accumulated during each epoch in a digital memory. These updates are blindly applied to the devices at the end of every epoch. However, directly applying the updates without accumulating is highly desirable to reduce additional digital memory requirements. To investigate it, we performed a training simulation where the weight updates are applied directly to the synapses at the precise instants of spike errors as illustrated in Fig. 4d. Not accumulating the updates leads to a larger number of smaller desired conductance changes in the synapses necessitating higher update precision and larger dynamic range for the synapses. Since the reliable range of conductance update from a single PCM is fixed, it was necessary to increase the number PCM devices per synapse to 32 to achieve experiment equivalent accuracy (Fig. 4e). We also implemented a weight refresh after every 13 epochs to avoid conductance saturation of the PCM devices from repeated SET programming. If the conductance of the positive half and the negative half of a synapse exceed 75% of their conductance range, all the PCMs in the synapse are reset. The original difference of conductance of the positive and the negative halves is converted to a number of programming pulses, based on the average conductance update granularity of the device, and 90 *μ*A, 50 ns programming pulses are applied in a cyclical manner to the PCMs in the synapse. This enabled the saturated synapses to further take part in training. Compared to the earlier accumulate and update scheme, spike-triggered updates demonstrated smaller fluctuations in spike time accuracy between training epochs. We analyzed the effect of conductance drift and device programming noise by turning them off in the model in separate training simulations (see Fig. 4f). The accuracy from spike-triggered synapse updates was less sensitive to the drift and noise since their absence only led to marginal improvements in the accuracy. For comparison, we also performed a spike-triggered synapse update based training using a 9-bit linear synapse (corresponding to 32 PCMs) which experienced more than 3% accuracy drop compared to the 7-bit linear synapse, which was programmed only at the end of each epoch based on accumulated updates. The observations here suggest that the precision and more frequent update errors originating from the non-linear and asymmetric update behavior limit the maximum accuracy when the devices are updated at each spike error.

### SNN inference using PCM synapses

**Figure 5:**
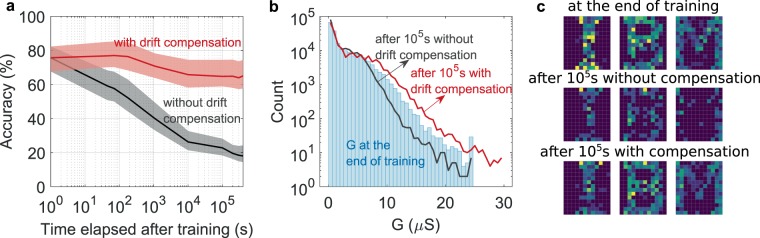
SNN inference using PCM synapses and drift compensation. (**a**) Spike-time accuracy as a function of time after training. The lower bound, middle line, and the upper bound indicate the percentage of spikes generated within 5 ms, 10 ms, and 25 ms tolerance interval, respectively. Due to conductance drift, the accuracy drops over time (black line). The effect of drift can be compensated by a time-aware scaling method (red line). Percentage accuracy drop over 4 × 10^5^ s was reduced from 70% to 13.6% at 25 ms error tolerance. (**b**) The distribution conductance drifted for 10^5^ s is compared with those immediately at the end of training. The distribution of conductance scaled to compensate for the effect of drift after 10^5^ s is also shown. (**c**) The images generated from the observed output spike streams from the SNN, immediately at the end of training (top), after 10^5^ s without drift compensation (middle), and after 10^5^ s with drift compensation (bottom). The brightness of each pixel represents the spike rate for the duration of each character.

In this section, we evaluate the ability of PCM to retain the trained state in an inference experiment. The PCM conductance values after the training experiment are read at logarithmic intervals in time and are used to determine the network response to the input spike streams used to train the network. In the absence of any compensation scheme, the spike time accuracy decreases over time as shown in Fig. 5a. The conductance distribution at the end of training and after 10^5^ s is shown in Fig. 5b. Due to the conductance drift, the net current flowing into the output neurons drop over time and output spikes begin to disappear. For example, the character images created based on the average number of spikes for each character duration immediately after the training and after 10^5^ s is shown in Fig. 5c, indicating a relative decay in the image quality. An array level scaling based on the knowledge of average drift coefficient and time elapsed since training has been shown to be effective in ANNs to counter the accuracy drop from PCM conductance drift^37^. We evaluate the effect of this approach to maintain spike time accuracy in the SNN here.

The conductance drift in the PCM is modeled using an empirical relation:

2 $$G(t)=G({t}_{0}){\left(\frac{t-{t}_{p}}{{t}_{0}-{t}_{p}}\right)}^{-\nu }$$

where *G*(*t*) is the conductance of the device at time $t > {t}_{p}$, *t*_*p*_ denotes the time when it received a programming pulse, *t*_0_ represents the time instant at which its conductance was last read after programming, and *v* is the drift coefficient^49^. Thus, each programming pulse effectively re-initializes the conductance drift^39^. As a result, the devices in the array will drift by different amounts during training, based on the instant they received the last weight update. However, once sufficient time has elapsed after training, (i.e. when *t* becomes much larger than all the *t*_*p*_ values for the devices in the array), the conductance drift can be compensated by an array level scaling. In our study, all the PCM conductances read over time from the prototype chip were scaled by ${t}_{e}^{0.035}$ where the *t*_*e*_ is the time elapsed since training and 0.035 is the effective drift coefficient for the conductance range of the devices in the array. In hardware, this can be implemented as a gain factor between the bit line currents from the crossbar array and the current integrated by the LIF neuron. Figure 5a shows that the spike time accuracy over time can be significantly improved using this compensation scheme. The scaled conductance distribution and its impact on the generated images from the output spike streams are shown in Fig. 5b,c, respectively. Note that the drift coefficient of the PCM demonstrates state dependency and hence more complex compensation schemes might be required in more advanced network architectures. Also, it is essential to reduce the conductance drift to further improve the inference performance of SNN using PCM synapses. A projected-PCM cell architecture proposed recently demonstrate an order of magnitude lower drift coefficient and is a promising step in this direction^50,51^.

## Discussion

Our work provides an experimental evaluation of the ability of phase-change memory devices to represent the synaptic strength in SNNs that have been trained to create spikes at precise time instances. The primary motivation here is to combine the energy benefits of in-memory computing hardware^9,34,35,52,53^ and sparse spike time based learning in SNN. Furthermore, spikes in biological neural networks are observed to be very precise in time^54–58^ and such spike time based computation is believed to be the source of computational efficiency in the human brain^59^.

As opposed to supervised learning in the second generation ANNs whose network outputs are determined typically by functions such as softmax, learning to generate multiple spikes at precise time instants is a harder problem. Compared to classification problems, the accuracy of which depends only on the relative magnitude of the response from one out of several output neurons, the task here is to generate close to 1000 spikes at the desired time instances over a period of 1250 ms from 168 spiking neurons, which are excited by 132 input spike streams. Our experimental demonstration is relevant to analog memory based neural network learning systems which attempts to make decisions based on the timing of a few spikes or generate precisely timed control signals for temporal actions as illustrated in Fig. 1a. Compared to the SNNs encoding information in the spike rate, spike time encoding could result in sparser computation and smaller response time^3,20,21,60,61^. SNNs are explored as an energy-efficient alternative to standard ANN based learning systems. However, the temporal dynamics of the SNN makes its implementation and training much more expensive in the conventional von Neumann architecture. Analog memory based non-von Neumann in-memory computing architecture provides dedicated connectivity between a large number of spiking neurons and real-time processing of asynchronous spike streams. So far, the experimental learning demonstrations using in-memory computing architectures have been limited to STDP and related Hebbian learning^30,32,62,63^. Our work is one of the first experimental evaluations of the ability of the neurons to generate and maintain spikes at precise time instances when the synapses are implemented using realistic analog memory devices.

The overall system efficiency is also dependent on circuits implementing functionalities such as neurons, weight updates, and core-to-core communication. While a dedicated circuit implementation for each of these functionalities may provide maximum operational parallelism, the circuit complexity, area benefits, and reconfiguration flexibility may foster a mixed digital-analog design for the peripheral computations. In our training experiment, the weight updates were accumulated in a digital memory before applying to the PCM devices. While it is desirable to avoid accumulating weight updates in a digital memory, the precision offered by the non-volatile analog memory devices alone is often not enough for a large class of learning problems. In this case, an analog-digital mixed-precision design could be more practical. Also, note that the number of spikes that is communicated through the synaptic memory arrays is typically much larger than the number of spikes that leads to a weight update. As a result, access to the digital memory could be relatively sparse and only a subset of this memory, corresponding to the synapses which receive the updates, is accessed during weight update accumulation. Hence, even the mixed analog-digital design has the potential to bring additional benefits with respect to performing the training fully in software. Further, computationally more expensive operations such as normalization in the NormAD training, which enables it to achieve high performance in floating point training, maybe simplified for the sake of hardware efficiency, if a more accurate learning circuit implementation does not lead to additional accuracy improvement in the presence of device non-idealities. Hence, the efficiency evaluation of such on-chip SNN learning systems require further software-hardware co-optimization research.

In summary, we analyzed the potential of PCM devices to realize synapses in SNNs that can learn to generate spikes at precise time instances via large scale (approximately 180,000 PCMs) supervised training and inference experiments and simulations. We successfully demonstrated that PCM based synapses of an SNN can be trained using incremental and stochastic updates to generate spikes at desired time instances within a few milliseconds of precision in a 1250 ms time window. We observe that recurring updates throughout the training (without an early stop) are beneficial in PCM based synapses which demonstrate conductance drift if the PCM programming noise can be reduced. Also, we show that the performance drop during inference due to the conductance drift could be compensated using a scaling methodology at the array periphery, based on a global factor which is a function of the time elapsed since training alone.

## Methods

### Audio to spike conversion

The silicon cochlea chip has 64 band-pass filters with frequency bands logarithmically distributed from 8 Hz to 20 kHz and generates spikes representing left and right channels. Further, due to the asynchronous delta modulation scheme used to create the spikes, there were on-spikes and off-spikes. The silicon cochlea generated spikes with a time resolution of 1 *μ*s. The spikes were further sub-sampled to a time resolution of 0.1 ms. The final input spike streams used for the training experiments have an average spike rate of 10 Hz. Combining all the filter responses with non-zero spikes for left and right channels and the on and off spikes, there are 132 input spike streams.

### Neuron model

The SNN output neurons were modeled using leaky-integrate and fire (LIF) model. Its membrane potential *V*(*t*) is given by the differential equation

$${C}_{m}\frac{dV(t)}{dt}=-{g}_{L}(V(t)-{E}_{L})+I(t)$$

where *C*_*m*_ is the membrane capacitance, *g*_*L*_ is the leak conductance, *E*_*L*_ is the leak reversal potential, and *I*(*t*) is the net synaptic current flowing into the neuron. When *V*(*t*) exceeds a threshold voltage *V*_*T*_, *V*(*t*) is reset to *E*_*L*_ and a spike is assumed to be generated. Once a spike is generated, the neuron is prevented from creating another spike within a short time period called refractory period *t*_*ref*_. For the training experiment, we used *C*_*m*_ = 300 pF, *g*_*L*_ = 30 nS, *E*_*L*_ = −70 mV, *V*_*T*_ = 20 mV, *t*_*ref*_ = 2 ms. For the NormAD training algorithm, the approximate impulse response of the LIF neuron is given as $\frac{1}{{C}_{m}}{e}^{-t/{\tau }_{L}}u(t)$ where ${\tau }_{L}=0.1\times {C}_{m}/{g}_{L}$ and $u(t)$ is the Heaviside step function. During training, the neuron responses were simulated using 0.1 ms time resolution.

### PCM platform

The experimental platform is built around a prototype chip of 3 million PCM cells. The PCM devices are based on doped-Ge_2_Sb_2_Te_5_ integrated in 90 nm CMOS technology^64^. The fabricated PCM cell area is 50 F^2^ (F is the feature size for the 90 nm technology node), and each memory device is connected to two parallel 240 nm wide n-type FETs. The chip has circuitry for cell addressing, ADC for readout, and circuits for voltage or current mode programming.

The PCM chip is interfaced with the Matlab workstation via FPGA boards and a high-performance analog-front-end (AFE) board. AFE implements digital to analog converters, electronics for power supplies, and voltage and current references. FPGA board implements digital logic for interfacing PCM and AFE board and perform data acquisition. A second FPGA board has an embedded processor and Ethernet unit for overall system control and data management.

### Experiment

The SNN training problem is initially simulated using double-precision (FP64) synapses in the Matlab simulation environment. The weight range for the SNN is approximately in the range [-6000, 6000]. To map the weights to the PCM conductance values in a multi-PCM configuration, the conductance range contribution from each device is assumed to be [0.1 *μ*S, 8 *μ*S]. For the training, the PCM devices in the hardware platform were randomly initialized to a conductance distribution of mean 0.66 *μ*S and standard deviation 0.53 *μ*S. The conductance values are read from the hardware using a constant read voltage of 0.3 V, scaled to the network weights and are used for matrix-vector multiplications in the software simulator. The weight updates determined from the training algorithm at the end of an epoch is programmed to the PCM devices using SET pulses of duration 50 ns and amplitudes in the range [40 *μ*A, 130 *μ*A]. The device conductance values are read after the update from the hardware and are used for the SNN synapse values for further training.

For inference, the PCM conductance values were read at logarithmic intervals in time after 100 epochs of training. To evaluate the inference performance in the absence of drift compensation, the values read from the chip are mapped to synaptic weights and are used to determine the SNN response. In the presence of the compensation scheme, the conductance values read from the prototype chip are first scaled using the global scaling factor based on the time elapsed since programming. This scaled conductance values are then mapped to weight values and is used to determine the SNN response.

## Supplementary information

Supplementary information.

## Data Availability

The data that support the plots within this paper and other findings of this study are available from the corresponding author upon reasonable request.

## References

[1] Lecun Y, Bengio Y, Hinton G (2015). Deep learning. Nature.

[2] Rajendran B, Sebastian A, Schmuker M, Srinivasa N, Eleftheriou E (2019). Low-power neuromorphic hardware for signal processing applications: A review of architectural and system-level design approaches. IEEE Signal Processing Magazine.

[3] Merolla PA (2014). A million spiking-neuron integrated circuit with a scalable communication network and interface. Science.

[4] Davies M (2018). Loihi: A Neuromorphic Manycore Processor with On-Chip Learning. IEEE Micro.

[5] Lichtsteiner P, Posch C, Delbruck T (2008). A 128 × 128 120 dB 15 *μ*s Latency Asynchronous Temporal Contrast Vision Sensor. IEEE Journal of Solid-State Circuits.

[6] Liu SC, Van Schaik A, Minch BA, Delbruck T (2014). Asynchronous binaural spatial audition sensor with 2 × 64 × 4 Channel output. IEEE Transactions on Biomedical Circuits and Systems.

[7] Sze, V., Chen, Y.-H., Yang, T.-J. & Emer, J. S. Efficient processing of deep neural networks: A tutorial and survey. *Proceedings of the IEEE* **105**, 2295–2329, arXiv:1703.09039 (2017).

[8] Burr GW (2017). Neuromorphic computing using non-volatile memory. Advances in Physics: X.

[9] Le Gallo M (2018). Mixed-precision in-memory computing. Nature Electronics.

[10] Sebastian A (2018). Tutorial: Brain-inspired computing using phase-change memory devices. Journal of Applied Physics.

[11] Sebastian, A., Le Gallo, M., Khaddam-Aljameh, R. & Eleftheriou, E. “Memory devices and applications for in-memory computing”, Nature Nanotechnology, 10.1038/s41565-020-0655-z (2020). 10.1038/s41565-020-0655-z 32231270

[12] Pfeiffer M, Pfeil T (2018). Deep learning with spiking neurons: Opportunities and challenges. Frontiers in Neuroscience.

[13] Cao, Y., Chen, Y. & Khosla, D. Spiking deep convolutional neural networks for energy-efficient object recognition. *International Journal of Computer Vision* **113**, 54–66, arXiv:1502.05777 (2015).

[14] Diehl, P. U., Zarrella, G., Cassidy, A., Pedroni, B. U. & Neftci, E. Conversion of artificial recurrent neural networks to spiking neural networks for low-power neuromorphic hardware, arXiv:1601.04187 (2016).

[15] Rueckauer B, Lungu I-A, Hu Y, Pfeiffer M, Liu S-C (2017). Conversion of continuous-valued deep networks to efficient event-driven networks for image classification. Frontiers in Neuroscience.

[16] Esser, S. K., Appuswamy, R., Merolla, P., Arthur, J. V. & Modha, D. S. Backpropagation for energy-efficient neuromorphic computing. In *Advances in Neural Information Processing Systems 28*, edited by Cortes, C. Lawrence, N. D. Lee, D. D. Sugiyama, M. & Garnett, R. (Curran Associates, Inc., 2015) pp. 1117–1125.

[17] Hunsberger, E. & Eliasmith, C. Training spiking deep networks for neuromorphic hardware. CoRR abs/1611.05141 (2016), arXiv:1611.05141.

[18] Woźniak, S., Pantazi, A. & Eleftheriou, E. Deep networks incorporating spiking neural dynamics. (2018), arXiv:1812.07040.

[19] Tavanaei A, Ghodrati M, Kheradpisheh SR, Masquelier T, Maida A (2019). Deep learning in spiking neural networks. Neural Networks.

[20] Bohte, S. M., Kok, J. N. & La Poutré, H. Error-backpropagation in temporally encoded networks of spiking neurons. *Neurocomputing* **48**, 17–37 (2002).

[21] Gütig R, Sompolinsky R (2006). The tempotron: a neuron that learns spike timing-based decisions. Nature neuroscience.

[22] Shrestha, S. B. & Orchard, G. Slayer: Spike layer error reassignment in time. In *Advances in Neural Information Processing Systems 31*, edited by Bengio, S. Wallach, H. Larochelle, H. Grauman, K. Cesa-Bianchi, N. & Garnett, R. (Curran Associates, Inc.) pp. 1412–1421 (2018).

[23] Ponulak F, Kasiski A (2010). Supervised learning in spiking neural networks with resume: Sequence learning, classification, and spike shifting. Neural Computation.

[24] Anwani, N. & Rajendran, B. Normad-normalized approximate descent based supervised learning rule for spiking neurons. In *International Joint Conference on Neural Networks (IJCNN)* (IEEE) pp. 1–8 (2015).

[25] Kulkarni SR, Rajendran B (2018). “Spiking neural networks for handwritten digit recognitionSupervised learning and network optimization,”. Neural Networks.

[26] Kuzum D, Jeyasingh RG, Lee B, Wong H-SP (2011). Nanoelectronic programmable synapses based on phase change materials for brain-inspired computing. Nano letters.

[27] Jackson BL (2013). Nanoscale electronic synapses using phase change devices. ACM Journal on Emerging Technologies in Computing Systems (JETC).

[28] Tuma T, Le Gallo M, Sebastian A, Eleftheriou E (2016). Detecting correlations using phase-change neurons and synapses. IEEE Electron Device Letters.

[29] Sidler, S., Pantazi, A., Woźniak, S., Leblebici, Y. & Eleftheriou, E. Unsupervised learning using phase-change synapses and complementary patterns. In *International Conference on Artificial Neural Networks* (Springer) pp. 281–288. (2017).

[30] Boybat I (2018). Neuromorphic computing with multi-memristive synapses. Nature Communications.

[31] Kim, S. *et al*. NVM neuromorphic core with 64k-cell (256-by-256) phase change memory synaptic array with on-chip neuron circuits for continuous in-situ learning. In *2015 IEEE International Electron Devices Meeting (IEDM)* pp. 17.1.1–17.1.4. (2015).

[32] Ambrogio S (2016). Unsupervised learning by spike timing dependent plasticity in phase change memory (PCM) synapses. Frontiers in Neuroscience.

[33] Burr GW (2015). Experimental demonstration and tolerancing of a large-scale neural network (165 000 synapses) using phase-change memory as the synaptic weight element. IEEE Transactions on Electron Devices.

[34] Ambrogio S (2018). Equivalent-accuracy accelerated neural-network training using analogue memory. Nature.

[35] Eleftheriou, E. *et al*. Deep learning acceleration based on in-memory computing. *IBM Journal of Research and Development*, 1–1 (2019).

[36] Oh S, Huang Z, Shi Y, Kuzum D (2019). The impact of resistance drift of phase change memory (PCM) synaptic devices on artificial neural network performance. IEEE Electron Device Letters.

[37] Ambrogio, S. *et al*. Reducing the impact of phase-change memory conductance drift on the inference of large-scale hardware neural networks. In *2019 IEEE International Electron Devices Meeting (IEDM)* (2019).

[38] Nandakumar, S. *et al*. Mixed-precision architecture based on computational memory for training deep neural networks. In *International Symposium on Circuits and Systems (ISCAS)* (IEEE) pp. 1–5 (2018).

[39] Boybat, I. *et al*. Impact of conductance drift on multi-PCM synaptic architectures. In *2018 Non-Volatile Memory Technology Symposium (NVMTS)* pp. 1–4 (2018).

[40] Suri, M. *et al*. Phase change memory as synapse for ultra-dense neuromorphic systems: Application to complex visual pattern extraction. In *Electron Devices Meeting (IEDM), 2011 IEEE International* pp. 4.4.1–4.4.4. (2011).

[41] Oh S, Shi Y, Liu X, Song J, Kuzum D (2018). Drift-enhanced unsupervised learning of handwritten digits in spiking neural network with PCM synapses. IEEE Electron Device Letters.

[42] Nomura, A. *et al*. NVM weight variation impact on analog spiking neural network chip. In *Neural Information Processing*, edited by L. Cheng, Leung, A. C. S. & Ozawa, S. (Springer International Publishing, Cham) pp. 676–685 (2018).

[43] Vianello, E. *et al*. Metal oxide resistive memory (OxRAM) and phase change memory (PCM) as artificial synapses in spiking neural networks. In *2018 25*^*th*^*IEEE International Conference on Electronics, Circuits and Systems (ICECS)* pp. 561–564 (2018).

[44] Close, G. F. *et al*. Device, circuit and system-level analysis of noise in multi-bit phase-change memory. In *IEEE International Electron Devices Meeting (IEDM)* (IEEE), pp. 29.5.1–29.5.4 (2010).

[45] Nandakumar S (2018). A phase-change memory model for neuromorphic computing. Journal of Applied Physics.

[46] Boniardi M (2009). A physics-based model of electrical conduction decrease with time in amorphous ge2sb2te5. Journal of Applied Physics.

[47] Boniardi M, Ielmini D (2011). Physical origin of the resistance drift exponent in amorphous phase change materials. Applied Physics Letters.

[48] Le Gallo M, Krebs D, Zipoli F, Salinga M, Sebastian A (2018). Collective structural relaxation in phase-change memory devices. Advanced Electronic Materials.

[49] Ielmini D, Sharma D, Lavizzari S, Lacaita AL (2009). Reliability impact of chalcogenide-structure relaxation in phase-change memory (PCM) cells-Part I: Experimental study. IEEE Transactions on Electron Devices.

[50] Koelmans WW (2015). Projected phase-change memory devices. Nature communications.

[51] Giannopoulos, I. *et al*. 8-bit Precision In-Memory Multiplication with Projected Phase-Change Memory. In *Proc. IEEE International Electron Devices Meeting (IEDM)*, pp. 27.7.1–27.7.4 (2018).

[52] Gokmen T, Vlasov Y (2016). Acceleration of Deep Neural Network Training with Resistive Cross-Point Devices: Design Considerations. Frontiers in Neuroscience.

[53] Song, L., Qian, X., Li, H. & Chen, Y. Pipelayer: A pipelined reram-based accelerator for deep learning. In *2017 IEEE International Symposium on High Performance Computer Architecture (HPCA)* pp. 541–552 (2017).

[54] Mainen Z, Sejnowski T (1995). Reliability of spike timing in neocortical neurons. Science.

[55] Bair W, Koch C (1996). Temporal precision of spike trains in extrastriate cortex of the behaving macaque monkey. Neural Computation.

[56] Berry, M. J., Warland, D. K., & Meister, M. The structure and precision of retinal spike trains. *Proceedings of the National Academy of Sciences* **94**, 5411–5416, https://www.pnas.org/content/94/10/5411.full.pdf (1997). 10.1073/pnas.94.10.5411 PMC24692 9144251

[57] Reich, D. S., Victor, J. D., Knight, B. W., Ozaki, T. & Kaplan, E. Response variability and timing precision of neuronal spike trains *in vivo*. *Journal of Neurophysiology* **77**, 2836–2841, pMID: 9163398, 10.1152/jn.1997.77.5.2836 (1997). 10.1152/jn.1997.77.5.2836 9163398

[58] Uzzell, V. J. & Chichilnisky, E. J. Precision of spike trains in primate retinal ganglion cells. *Journal of Neurophysiology* 92, 780–789, 10.1152/jn.01171.2003, pMID: 15277596 (2004). 10.1152/jn.01171.2003 15277596

[59] Maass W (1997). Noisy spiking neurons with temporal coding have more computational power than sigmoidal neurons. Advances in Neural Information Processing Systems.

[60] Crotty P, Levy WB (2005). Energy-efficient interspike interval codes. Neurocomputing.

[61] Wang B (2016). Firing frequency maxima of fast-spiking neurons in human, monkey, and mouse neocortex. Frontiers in Cellular Neuroscience.

[62] Eryilmaz SB (2014). Brain-like associative learning using a nanoscale non-volatile phase change synaptic device array. Frontiers in Neuroscience.

[63] Sebastian A (2017). Temporal correlation detection using computational phase-change memory. Nature Communications.

[64] Breitwisch, M. *et al*. Novel lithography-independent pore phase change memory. In *IEEE Symposium on VLSI Technology* (IEEE) pp. 100–101 (2007).

