# Supplementary material for: Experimental Demonstration of Supervised Learning in Spiking Neural Networks with Phase-Change Memory Synapses
Source: Sci Rep. 2020 May 15;10:8080. doi: 10.1038/s41598-020-64878-5 (PMC7228943; doi:10.1038/s41598-020-64878-5)
Supplement: Supplementary file 1 — Supplementary information. [file 41598_2020_64878_MOESM1_ESM.pdf]

Experimental Demonstration of Supervised Learning in Spiking Neural Networks with Phase-Change Memory Synapses

S. R. Nandakumar,[1,2] Irem Boybat,[1,3] Manuel Le Gallo,[1] Evangelos Eleftheriou,[1] Abu Sebastian,[1,a] and Bipin Rajendran[4,b]

[1] *IBM Research – Zurich, 8803 Rüschlikon, Switzerland*
[2] *New Jersey Institute of Technology, Newark, NJ 07102, USA*
[3] *Ecole Polytechnique Federale de Lausanne (EPFL), 1015 Lausanne, Switzerland*
[4] *King's College London, Strand, London WC2R 2LS, United Kingdom*

(Dated: 6 May 2020)

---

[a] Electronic mail: ase@zurich.ibm.com
[b] Electronic mail: bipin.rajendran@kcl.ac.uk

SUPPLEMENTARY FIGURE 1: CONDUCTANCE DISTRIBUTION FROM SNN TRAINING EXPERIMENT AND PCM MODEL

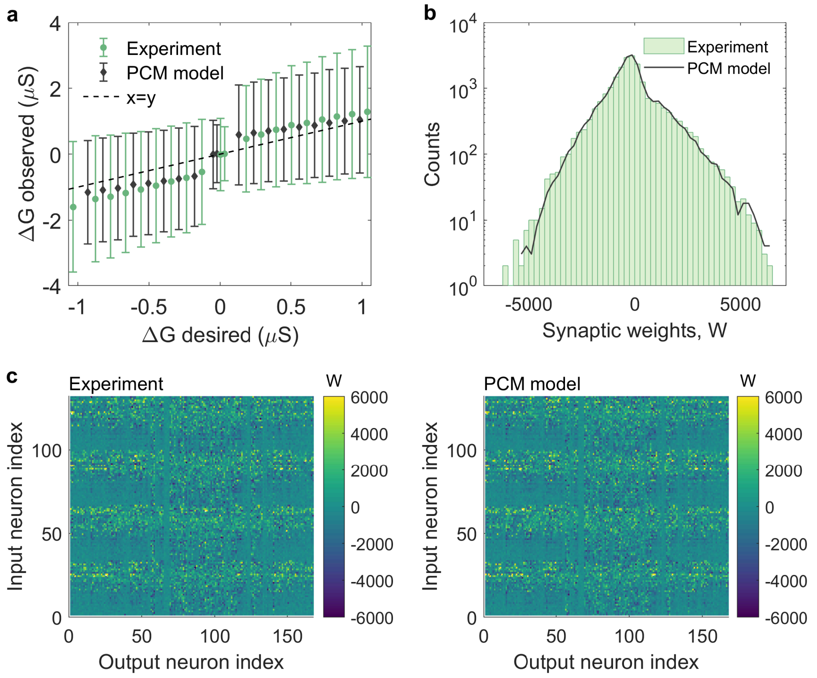

Supplementary Fig. 1. **Conductance distribution from SNN training experiment and PCM model.** **a** The observed versus desired conductance change (ΔG) from the phase-change memory (PCM) device used in the SNN training experiment. Corresponding response from the training simulation using PCM model is shown for comparison. **b** Weight distribution observed at the end of the training experiment using the PCM devices and those from the PCM model based simulation. **c** Illustration of the synaptic weights from the PCM device based training experiment and the PCM model based training simulation. It can be seen that the model reliably captures the experimental behavior.

SUPPLEMENTARY NOTE 1: PCM TEMPORAL DYNAMICS

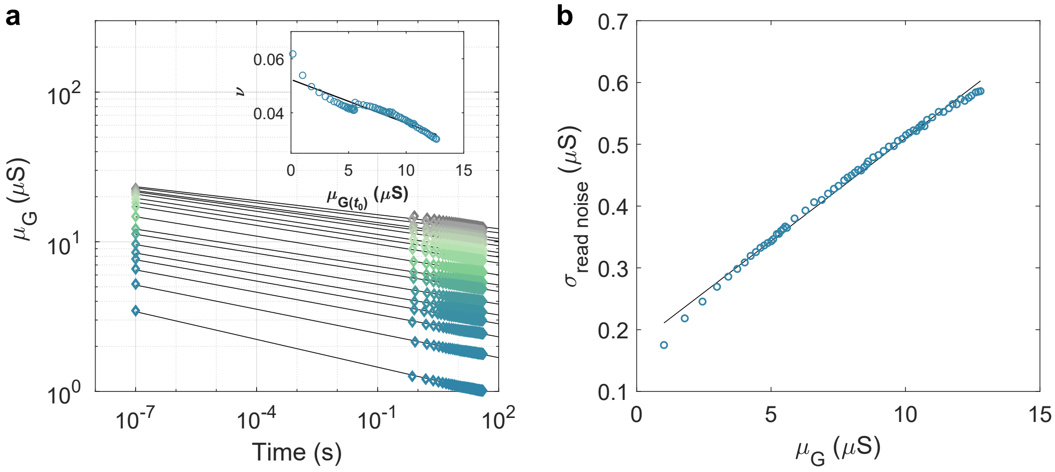

Supplementary Fig. 2. **PCM temporal dynamics. a** The temporal drift observed in the average conductance (μ_G) after SET pulse programming. The estimated drift coefficients as a function of the conductance is shown in the inset. **b** The standard deviation of read noise ($\sigma_{\text{read noise}}$) measured as a function of the average PCM conductance.

PCM exhibits read noise and conductance drift over time. In this note we describe the model fit lines used to represent state-dependent drift coefficient and state-dependent standard deviation of read noise. 10,000 PCM devices were iteratively programmed to a distribution around $0.1\,\mu$S and were subjected to 20 SET pulses of $90\,\mu$A amplitude and 50 ns duration. The programming sequence gradually increases the device conductance. The programmed states were read 50 times. The average conductance evolution over time t during the read events is well captured using an empirical relation given by

$$G(t) = G(t_0)\left(\frac{t-t_p}{t_0-t_p}\right)^{-\nu}, \qquad (1)$$

where $G(t_0)$ is the measured conductance at time t_0 after a programming event at time t_p, and ν is the drift coefficient[1–3]. The experimentally measured evolution of the mean conductance of 10,000 PCM devices are fitted using Equation (1) (Supplementary Fig. 2a). The extracted drift coefficients decrease with increasing conductance values as shown in the inset. For the training simulations, the drift coefficients are estimated using the expression, $\nu = -0.0016 \times G(t_0) + 0.05$, where the conductance values are in micro Siemens and $t_0 = 41.47$s.

The last 10 reads, after each SET pulse, during which the drift is negligible are used to estimate the standard deviation of read noise ($\sigma_{\text{read noise}}$). The standard deviation averaged from the 10,000 devices is plotted as function of the average conductance (Supplementary Fig. 2b). For the PCM based training simulations, the read noise is modeled as a zero mean Gaussian distribution whose standard deviation increases with the device conductance according to the fit line, $\sigma_{\text{read noise}} = 0.03 \times G + 0.17$.

[1] A. Pirovano, A. Lacaita, F. Pellizzer, S. Kostylev, A. Benvenuti, and R. Bez, "Low-field amorphous state resistance and threshold voltage drift in chalcogenide materials," IEEE Transactions on Electron Devices **51**, 714–719 (2004).
[2] D. Ielmini, D. Sharma, S. Lavizzari, and A. L. Lacaita, "Reliability impact of chalcogenide-structure relaxation in phase-change memory (PCM) cells-Part I: Experimental study," IEEE Transactions on Electron Devices **56**, 1070–1077 (2009).
[3] M. Le Gallo, D. Krebs, F. Zipoli, M. Salinga, and A. Sebastian, "Collective structural relaxation in phase-change memory devices," Advanced Electronic Materials **4**, 1700627 (2018).
